# Identification of Novel Rosavirus Species That Infects Diverse Rodent Species and Causes Multisystemic Dissemination in Mouse Model

**DOI:** 10.1371/journal.ppat.1005911

**Published:** 2016-10-13

**Authors:** Susanna K. P. Lau, Patrick C. Y. Woo, Kenneth S. M. Li, Hao-Ji Zhang, Rachel Y. Y. Fan, Anna J. X. Zhang, Brandon C. C. Chan, Carol S. F. Lam, Cyril C. Y. Yip, Ming-Chi Yuen, Kwok-Hung Chan, Zhi-Wei Chen, Kwok-Yung Yuen

**Affiliations:** 1 State Key Laboratory of Emerging Infectious Diseases, The University of Hong Kong, Hong Kong, China; 2 Research Centre of Infection and Immunology, The University of Hong Kong, Hong Kong, China; 3 Carol Yu Centre for Infection, The University of Hong Kong, Hong Kong, China; 4 Department of Microbiology, The University of Hong Kong, Hong Kong, China; 5 Department of Veterinary Medicine, Foshan University, Foshan, China; 6 Food and Environmental Hygiene Department, Hong Kong, China; Blood Systems Research Institute, UNITED STATES

## Abstract

While novel picornaviruses are being discovered in rodents, their host range and pathogenicity are largely unknown. We identified two novel picornaviruses, rosavirus B from the street rat, Norway rat, and rosavirus C from five different wild rat species (chestnut spiny rat, greater bandicoot rat, Indochinese forest rat, roof rat and Coxing's white-bellied rat) in China. Analysis of 13 complete genome sequences showed that “Rosavirus B” and “Rosavirus C” represent two potentially novel picornavirus species infecting different rodents. Though being most closely related to rosavirus A, rosavirus B and C possessed distinct protease cleavage sites and variations in Yn-Xm-AUG sequence in 5’UTR and myristylation site in VP4. Anti-rosavirus B VP1 antibodies were detected in Norway rats, whereas anti-rosavirus C VP1 and neutralizing antibodies were detected in Indochinese forest rats and Coxing's white-bellied rats. While the highest prevalence was observed in Coxing's white-bellied rats by RT-PCR, the detection of rosavirus C from different rat species suggests potential interspecies transmission. Rosavirus C isolated from 3T3 cells causes multisystemic diseases in a mouse model, with high viral loads and positive viral antigen expression in organs of infected mice after oral or intracerebral inoculation. Histological examination revealed alveolar fluid exudation, interstitial infiltration, alveolar fluid exudate and wall thickening in lungs, and hepatocyte degeneration and lymphocytic/monocytic inflammatory infiltrates with giant cell formation in liver sections of sacrificed mice. Since rosavirus A2 has been detected in fecal samples of children, further studies should elucidate the pathogenicity and emergence potential of different rosaviruses.

## Author Summary

We identified two novel picornaviruses, rosavirus B and C, infecting street and wild rats respectively in China. While rosavirus B was detected from Norway rats, rosavirus C was detected from five different wild rat species (chestnut spiny rat, greater bandicoot rat, Indochinese forest rat, roof rat and Coxing's white-bellied rat) by RT-PCR. Anti-rosavirus B antibodies were detected in Norway rats, whereas anti-rosavirus C antibodies were detected in Indochinese forest rats and Coxing's white-bellied rats, supporting potential interspecies transmission of rosavirus C. Genome analysis supported the classification of rosavirus B and C as two novel picornavirus species, with genome features distinct from rosavirus A. Rosavirus C isolated from 3T3 cells causes multisystemic diseases in a mouse model, with viruses and pathologies detected in various organs of infected mice after oral or intracerebral inoculation. Our results extend our knowledge on the host range and pathogenicity of rodent picornaviruses.

## Introduction

Picornaviruses are positive-sense, single-stranded RNA viruses with icosahedral capsids. They infect various animals and human, causing various respiratory, cardiac, hepatic, neurological, mucocutaneous and systemic diseases [1, 2]. Based on genotypic and serological characterization, the family *Picornaviridae* is currently divided into 29 genera with at least 50 species. Among the various picornaviruses belonging to nine genera that are able to infect humans, poliovirus and human enterovirus A71 are best known for their neurotropism and ability to cause mass epidemics with high morbidities and mortalities [3, 4]. Picornaviruses are also known for their potential for mutations and recombination, which may allow the generation of new variants to emerge [5–10].

Emerging infectious diseases like avian influenza and coronaviruses have highlighted the impact of animal viruses after overcoming the inter-species barrier [11–15]. As a result, there has been growing interest to understand the diversity and evolution of animal and zoonotic viruses. For picornaviruses, numerous novel human and animal picornaviruses have been discovered in the past decade [1, 16–27]. We have also discovered a novel picornavirus, canine picodicistrovirus (CPDV), with two internal ribosome entry site (IRES) elements, which represents a unique feature among *Picornaviridae* [28]. Moreover, novel picronaviruses were identified in previously unknown animal hosts such as cats, bats and camels [29–31], reflecting our slim knowledge on the diversity and host range of picornaviruses. The discovery and characterization of novel picornaviruses is important for better understanding of their evolution, pathogenicity and emergence potential.

Although rodents can be infected by several picornaviruses, the picornaviral diversity is probably underestimated, given the enormous species diversity of rodents. Moreover, little is known about the pathogenicity of the recently discovered rodent pricornaviruses, such as rodent stool-associated picornavirus (rosavirus) A1, mouse stool-associated picornavirus (mosavirus) A1, Norway rat hunnivirus and rat-borne virus (rabovirus A) [32, 33]. In this report, we explored the diversity of picornaviruses among rodents in China and discovered two potentially novel picornaviruses, “Rosavirus B” and “Rosavirus C”. While rosavirus B was detected in the street rat, Norway rats, rosavirus C was detected in five different wild rat species, suggesting potential interspecies transmission. Their complete genome sequences were determined, which showed that “Rosavirus B” and “Rosavirus C” represent two novel picornavirus species distinct from *Rosavirus A*. Rosavirus C isolated from cell culture causes multisystemic diseases in a mouse model, with histopathological changes and positive viral antigen expression in lungs and liver of infected mice.

## Results

### Identification of novel rodent picornaviruses, rosavirus B and C

**Fig 1:**
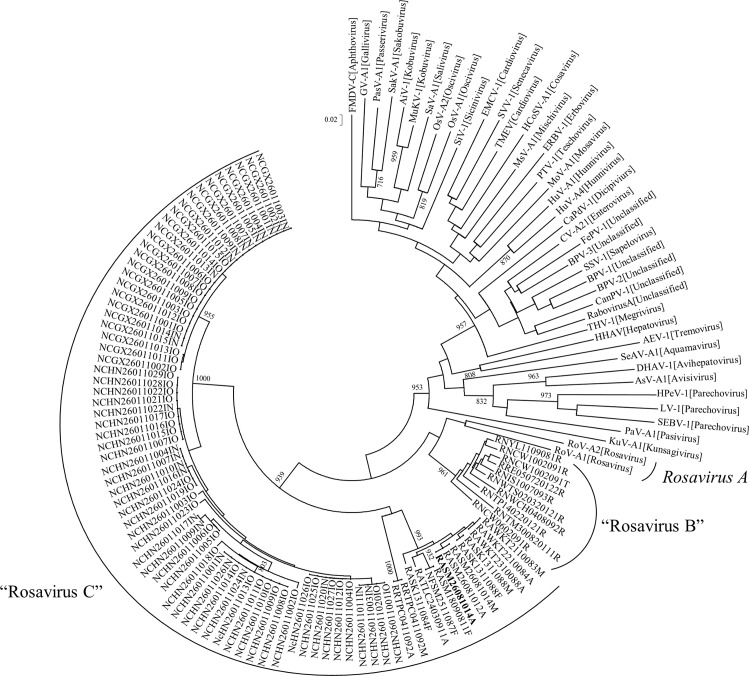
Phylogenetic analysis of nucleotide sequences of the 80-bp fragment (excluding primer sequences) of 3D^pol^ gene of a picornavirus identified from rodents in this study. The strain RASM14A that was successfully isolated in 3T3 cells is in bold. The trees were constructed by neighbor-joining method with bootstrap values calculated from 1000 trees. Only values greater than 700 are indicated. The scale bar indicates the estimated number of substitutions per 50 nucleotides.

A total of 2450 respiratory and alimentary samples from 1232 rodents of six different species were obtained (Table 1). Initial screening by RT-PCR for a 159-bp fragment of 3D^pol^ gene of picornaviruses was positive in 18 respiratory and 24 alimentary samples from 37 rodents of six different species. The sequences from these positive samples had <84% aa identities to the corresponding segments of 3D^pol^ genes of known picornaviruses, suggesting the presence of potential novel picornaviruses. Subsequent RT-PCR using specific primers targeting these potential novel picornaviruses on the 2450 samples was positive in 62 respiratory and 70 alimentary samples from 92 rodents. The sequences from these 132 samples had <86% aa identities to the corresponding segments of 3D^pol^ genes of known picornaviruses, being most closely related to *Rosavirus A*. Moreover, these sequences fell into two distinct clusters, one formed by sequences from wild rodents of five different species from Hong Kong (chestnut spiny rat, greater bandicoot rat, Indochinese forest rats and roof rat) and Hunan and Guangxi (Coxing's white-bellied rat), and the other formed by sequences from street rodents from Hong Kong (Norway rat) (Table 1 and Fig 1). This suggested the presence of two potentially novel picornavirus species, “Rosavirus B” and “Rosavirus C.”

**Table 1:** Rodents tested for picornaviruses in the present study.

Scientific name	Common name	No. of rodents positive/tested (%) for picornavirus in respiratory sample by RT-PCR	No. of rodents positive/tested (%) for picornavirus in alimentary sample by RT-PCR	Picornavirus species detected by sequencing	No. of rodents positive/tested (%) for respective picornavirus antibody by VP1-based western blot assay
*Bandicota indica*	Greater bandicoot rat	0/1 (0%)	1/1 (100%)	“Rosavirus C”	NA
*Niviventer coxingi*	Coxing's white-bellied rat	42/44 (95.5%)	25/44 (56.8%)	“Rosavirus C”	3/40 (7.5%)
*Niviventer fulvescens*	Chestnut spiny rat	3/205(1.5%)	5/206 (2.4%)	“Rosavirus C”	NA
*Rattus andamanensis*	Indochinese forest rat	11/457(2.4%)	16/458 (3.4%)	“Rosavirus C”	3/70 (4.3%)
*Rattus norvegicus*	Norway rat	5/458 (1.1%)	20/458 (4.4%)	“Rosavirus B”	2/61 (3.3%)
*Rattus rattus*	Roof rat	1/59 (1.7%)	3/59 (5.1%)	“Rosavirus C”	NA

Viral sequences belonging to “Rosavirus B” were only detected from the street rodent species, Norway rat (*Rattus norvegicus*), whereas sequences belonging to “Rosavirus C” were detected from five different wild rodent species, greater bandicoot rat (*Bandicota indica*), chestnut spiny rat (*Niviventer fulvescens*), roof rat (*Rattus rattus*) and Indochinese forest rat (*Rattus andamanensis*) from Hong Kong, and Coxing’s white-bellied rat (*Niviventer coxingi*) from Hunan and Guangxi. Among the five wild rodent species, rosavirus C showed the highest detection rate in Coxing’s white-bellied rats. qRT-PCR showed that the viral load of rosavirus B and C in the positive samples ranged from 1.7 ×10^4^ to 1.6×10^8^ copies/ml.

### Genome organization and coding potential of rosavirus B and C

A total of 13 complete genomes from samples of four different wild rodent species (chestnut spiny rat, Coxing’s white-bellied rat, roof rat and Indochinese forest rat) positive for “Rosavirus C” and one street rodent species (Norway rat) positive for “Rosavirus B” were sequenced directly from the positive respiratory or alimentary samples and characterized. These 13 strains were selected because they were detected from different rodent species or geographical locations (Hong Kong, Hunan and Guangxi) to allow comparison between host species- or geographically distinct strains. The G + C contents of the three rosavirus B and 10 rosavirus C genomes range from 50 to 53%, with genome size 8639 to 9094 bases, after excluding the polyadenylated tract (Table 2). However, the genome sizes of some strains may be larger, as further sequencing of the ends may have been hampered by secondary structures. They share similar genome organization typical of picornaviruses, with UTR at both 5' (470–752 bases) and 3' (693–948 bases) ends, and a large open reading frame of 7449–7476 bases, which encodes potential polyprotein precursors of 2482–2491 aa known to be cleaved by virus-encoded proteases. The predicted protease cleavage sites at P1 (encoding capsid proteins) P2 and P3 (both encoding non-structural proteins) are shown in Table 3. Notably, “Rosavirus B” differed from *Rosavirus A* in VP2/VP3 (P1’), VP3/VP1 (P1’), VP1/2A (P1’) and 2C/3A (P1’) cleavage sites, whereas “Rosavirus C” differed from *Rosavirus A* in VP4/VP2 (P1’), VP2/VP3 (P1’), VP3/VP1 (P1 and P1’), VP1/2A (P1’), 2A/2B (P1’), 2B/2C (P1’) and 2C/3A (P1’) cleavage sites.

**Table 2:** Comparison of amino acid identities between the predicted proteins P1, P2 and P3 of “Rosavirus B” and “Rosavirus C” and those of other representative picornavirus species.

genus	Species (type)	Size (bases)	G + C content	5’UTR (ntd)	P1 (aa)	P2^a^ (aa)	P3^b^ (aa)	3’UTR (ntd)	5’UTR (ntd)	P1 (aa)	P2^a^ (aa)	P3^b^ (aa)	3'UTR (ntd)
*Apthovirus*	*Foot-and-mouth disease virus*	8115	0.54	31.0–31.8	22.2–22.4	25.7–27.0	27.7–27.9	8.5–11.7	34.8–35.7	21.6–22.8	24.0–26.1	27.4–28.7	8.3–11.5
*Aquamavirus*	*Aquamavirus A*	6702	0.44	44.7–47.1	19.4–19.9	22.8–22.9	23.2–23.3	3.4–4.5	44.8–47.2	19.1–20.6	21.2–22.9	22.4–24.0	3.2–4.7
*Avihepatovirus*	*Avihepatovirus A*	7687	0.43	43.9–45.5	17.3–17.5	23.0–23.8	23.7–23.9	25.1–33.1	45.4–47.3	17.9–19.1	20.4–21.7	23.6–24.8	24.7–32.2
*Avisivirus*	*Avisivirus A*	7532	0.45	45.1–46.9	19.5–20.8	25.0–25.3	25.5–26.1	21.1–27.8	45.2–47.5	17.3–19.1	22.0–25.1	24.2–24.7	21.1–27.3
*Cardiovirus*	*Cardiovirus A*	7835	0.49	35.3–37.5	23.3–23.5	25.7–25.9	29.0–29.6	11.2–14.9	39.6–41.9	22.7–23.4	24.6–25.9	30.4–32.0	10.9–14.6
	*Cardiovirus B*	8101	0.49	31.2–31.6	24.4–24.7	26.5–28.2	28.6	10.7–14.6	32.4–34.8	23.4–24.0	23.2–25.3	28.7–30.8	10.9–14.9
*Cosavirus*	*Cosavirus A*	7632	0.44	27.7–29.4	22.8–23.2	26.2	28.3–28.5	8.2–11.7	32.0–33.6	22.9–24.7	24.5–26.2	27.6–29.7	8.1–11.5
*Dicipivirus*	*Cadicivirus A*	8755	0.42	32.9–33.1	34.7–35.0	28.8–29.4	41.9–42.2	38.5–44.1	35.9–37.5	34.6–35.7	25.6–28.3	42.9–44.5	37.9–44.5
*Enterovirus*	*Enterovirus C*	7401	0.45	40.4–41.5	21.0–22.2	23.2	29.1–29.4	6.8–9.2	41.7–43.8	20.4–22.9	21.7–23.2	28.9–29.8	6.8–8.8
*Erbovirus*	*Erbovirus A*	8828	0.49	37.3–37.6	22.4–23.4	23.3–23.6	27.7–27.9	13.9–19.0	41.6–43.0	22.4–23.8	21.6–23.1	26.4–28.3	14.6–19.0
*Gallivirus*	*Gallivirus A*	8496	0.48	39.5–41.0	20.0–20.2	27.1–27.6	35.0–35.2	24.8–31.6	42.4–45.0	18.5–20.8	27.1–28.8	34.6–36.1	25.2–31.9
*Hepatovirus*	*Hepatovirus A*	7478	0.38	40.0–40.5	20.2–20.8	25.2–25.4	25.7–26.1	5.4–7.5	41.2–43.5	19.6–20.9	22.7–24.4	25.1–26.2	5.6–8.2
*Hunnivirus*	*Hunnivirus A* (HuV-A1)	7583	0.46	41.0–41.8	23.2–23.5	24.5–25.0	28.3–28.7	11.2–14.1	46.0–48.4	21.6–23.2	24.4–25.3	30.0–31.0	11.0–15.0
	*Hunnivirus A* (HuV-A4)	7496	0.48	46.7–47.6	22.5–23.3	24.7–25.0	27.6	1.1–1.6	51.3–54.4	24.5–25.5	23.4–24.9	29.7–30.6	1.1–1.6
*Kobuvirus*	*Aichivirus A* (AiV-1)	8251	0.59	41.2–41.9	22.9–23.1	29.6	34.2–34.5	19.9–25.8	42.4–43.6	23.0–24.1	27.6–29.1	32.5–33.7	19.3–25.0
	*Aichivirus A* (MuKV-1)	8171	0.57	43.1–44.4	23.6–23.8	28.3–28.9	34.6–34.8	20.3–26.0	44.2–47.2	21.4–23.4	27.8–28.8	33.3–34.2	20.1–25.8
*Kunsagivirus*	*Kunsagivirus A*	7272	0.53	44.9–46.4	20.3–20.9	21.3	23.6–24.0	2.1–3.2	45.9–47.8	19.3–20.4	21.9–23.6	23.8–24.8	2.6–3.5
*Megrivirus*	*Melegrivirus A*	9075	0.46	47.9–48.2	20.6–21.0	30.8	37.8–38.1	14.8–19.9	44.1–46.5	21.0–22.5	29.7–31.5	35.1–36.3	14.7–20.1
*Mischivirus*	*MischivirusA*	8457	0.48	24.8–25.2	22.5–23.1	26.2–26.5	29.6–29.8	18.1–23.1	27.2–28.7	23.6–24.9	26.7–27.9	29.7–30.9	18.2–23.0
*Mosavirus*	*Mosavirus A*	6934	0.45	21.9–23.5	22.5–22.9	24.7–25.4	28.2–28.6	8.2–11.4	19.8–21.3	21.8–23.6	23.6–25.9	28.6–29.7	8.1–11.5
*Oscivirus*	*Oscivirus A* (OsV-A1)	7625	0.47	42.7–43.9	20.4–20.7	27.9–28.1	37.7–37.9	17.6–24.2	43.8–46.0	21.3–22.4	29.3–30.2	36.3–37.0	17.9–23.2
	*Oscivirus A* (OsV-A2)	7663	0.47	43.5–43.7	20.7–21.1	28.0–28.1	37.6–37.7	19.7–25.4	43.5–47.4	20.1–22.1	28.7–29.6	35.9–37.5	19.6–24.6
*Parechovirus*	*Parechovirus A*	7329	0.40	45.5–45.9	18.1–18.4	23.7–24.1	22.4–22.8	8.3–11.4	45.9–49.0	19.5–20.9	20.2–21.4	23.3–25.1	7.9–11.2
	*Parechovirus B*	7590	0.43	44.6–45.2	18.1–18.2	25.5–25.7	25.1–25.4	10.0–13.0	48.9–50.8	18.9–20.4	22.9–25.0	25.3–26.7	9.9–13.6
	Unclassified (SEBV-1)	7537	0.46	42.5–43.8	19.3–19.7	25.0–25.3	25.1–25.7	7.6–10.4	45.0–46.6	19.4–20.6	23.3–25.4	25.6–26.5	7.3–10.2
*Pasivirus*	*Pasivirus A*	6896	0.43	43.3–43.9	18.5–18.9	26.0–26.2	24.2–24.3	10.5–14.0	39.3–43.6	19.0–20.4	22.8–23.9	23.7–24.6	10.4–14.0
*Passerivirus*	*Passerivirus A*	8019	0.58	44.7–45.1	21.3–21.9	26.5–26.7	34.7–35.0	25.8–32.0	41.4–44.4	20.9–22.0	28.4–31.2	33.4–35.0	26.6–32.3
*Rosavirus*	*Rosavirus A* (RoV-A1)	8724	0.52	61.6–62.0	54.7–55.0	58.9–59.3	67.5–67.6	50.7–52.1	58.3–60.8	51.1–52.3	51.1–52.0	64.4–65.9	46.7–49.1
	*Rosavirus A* (RoV-A2)	8931	0.51	40.6–41.0	54.3–54.5	58.3–58.7	67.9–68.0	49.7–51.3	45.0–47.6	51.5–52.5	52.4–54.2	64.0–66.1	44.7–48.1
	“Rosavirus B”	8639–8923	0.51	95.1–95.7	95.7–98.4	98.5–99.4	99.1–99.0	70.7–98.1	58.4–61.9	57.7–59.4	58.5–61.1	68.4–70.4	43.6–55.0
	“Rosavirus C”	8697–9094	0.50–0.53	58.4–61.9	57.7–59.4	58.5–61.1	68.4–70.4	43.6–55.0	69.5–100	83.8–100	84.3–99.8	87.2–99.9	55.9–99.9
*Sakobuvirus*	*Sakobuvirus A*	7807	0.56	44.5–45.5	22.0–22.3	27.5	33.9–34.2	14.0–17.3	46.8–48.9	21.6–23.4	26.9–28.4	32.8–33.4	13.3–18.1
*Salivirus*	*Salivirus A*	7982	0.57	40.3–41.8	21.9–22.4	30.7–30.9	33.6–33.7	12.5–17.3	42.0–43.6	22.1–23.7	29.0–30.1	32.3–33.5	13.0–17.3
*Sapelovirus*	*Sapelovirus B*	8126	0.40	39.6–40.6	22.9–23.2	24.2–24.4	30.9–31.1	8.9–11.0	43.0–44.2	24.6–25.3	23.0–24.4	31.4–32.7	9.2–11.9
*Senecavirus*	*Senecavirus A*	7280	0.52	42.2–43.7	22.6–23.9	26.6–27.6	30.8–30.9	6.6–8.4	42.9–45.6	22.1–23.1	24.9–26.8	28.8–29.8	6.5–8.9
*Sicinivirus*	*Sicinivirus A*	9243	0.54	40.8–42.4	21.1–21.4	29.3–29.5	34.5–35.0	21.7–29.1	37.7–40.0	21.5–22.6	27.7–29.7	33.8–35.6	22.0–28.6
*Teschovirus*	*Teschovirus A*	7110	0.45	42.4–42.9	21.5–22.2	22.5–23.0	29.4–29.8	5.9–8.1	38.6–40.0	22.8–23.1	23.3–24.5	30.3–30.9	6.4–8.6
*Tremovirus*	*Tremovirus A*	7032	0.45	47.2–47.5	22.1–22.6	22.9–24.3	25.9–26.7	11.7–15.7	44.7–46.6	21.5–23.3	23.5–25.0	26.3–27.2	11.3–15.7
Unclassified	Unclassified (Bat picornavirus 1)	7737	0.45	44.1–46.2	23.6–24.2	25.3–25.7	30.4–30.5	15.2–20.1	41.5–44.4	25.3–26.1	22.3–23.6	30.3–31.2	15.1–19.7
	Unclassified (Bat picornavirus 2)	7677	0.43	42.3–42.8	24.1–24.2	24.0–24.3	27.6–27.9	12.2–16.0	40.9–44.0	24.4–24.9	22.7–23.3	29.8–30.9	12.4–16.3
	Unclassified (Bat picornavirus 3)	7731	0.50	45.9–47.4	23.6–24.2	25.7–26.3	28.9–29.3	18.2–24.1	43.9–46.6	24.2–25.9	24.5–25.1	28.8–30.2	18.2–23.8
	Unclassified (Canine picornavirus 1)	7948	0.41	39.6–41.2	23.8–24.1	22.8–23.0	28.6–28.9	12.7–16.9	43.6–45.0	23.5–24.7	23.5–25.4	28.9–29.6	13.2–17.3
	Unclassified (Feline picornavirus 1)	7415	0.50	40.3–41.8	24.0–24.6	25.3–25.7	28.8–29.3	7.2–10.1	37.3–39.5	23.9–25.0	24.8–26.2	30.1–31.4	7.3–10.0
	Unclassified (Rabovirus A)	7834	0.43	40.1–40.6	27.0–27.5	24.5–24.6	30.6–31.0	7.2–9.2	44.6–46.4	24.4–25.6	23.2–23.9	29.1–30.8	7.0–9.5

^a^P2 region excluding 2A

^b^P3 region excluding 3A

**Table 3:** Coding potential and putative proteins of “Rosavirus B” and “Rosavirus C” compared to *Rosavirus A*.

Putative protein	*Rosavirus A*		“Rosavirus B”		“Rosavirus C”	
	Position (P1-P1’)	Length (aa)	Position (P1-P1’)	Length (aa)	Position (P1-P1’)	Length (aa)
P1						
VP4	M_1_-N_57_	57	M_1_-N_58_	58	M_1_-N_59_	59
VP2	D/S_58_-E/Q_314_	257	S_59_-Q_314_	256	D/**N**_60_-E_312_	253
VP3	S_315_-E_589/593_	275/279	**N**_315_-E_593_	279	**G**_313_-**Q**_609/610_	297/298
VP1	H_590/594_-E_863_/Q_866_	274/273	**G**_594_-Q_862_	269	**V**/**A**_610_/**S**_611_-Q_876_	266/267
P2						
2A	L_864/867_-W_1135/1132_	272/266	**Y**_863_-W_1165_	303	L/**M**_877_-W_1155/1158_	279/282
2B	L_1136_/I_1133_-E_1312_	177/180	I_1166_-E_1345_	180	**T**_1156/1159_-E_1342/1343/1345/1346_	187/188/190
2C	A/S_1313_-E_1660_	348	S_1346_-E_1689_	344	**K**_1343/1344/1346/1347_-E_1687/1688/1689/1691_	344/345
P3						
3A	A_1661_-E_1755/1753_	95/93	**N**_1690_-E_1787_	98	**N**_1688/1689/1690/1692_-E_1778/1779/1780/1782_	91
3B	G_1756/1754_-E_1782/1780_	27	G_1788_-E_1814_	27	G_1779/1780/1781/1783_-E_1804/1805/1806/1808_	26
3C	G_1783/1781_-E_1986/1983_	204/203	G_1815_-E_2014_	200	G_1805/1806/1807/1809_-E_2004/2005/2006/2008_	200
3D	G_1987/1984_-Q_2470/2468_	484/485	G_2015_-**A**_2491_	477	G_2005/2006/2007/2009_-**V**_2482/2483/2484/2486_	478

Residues at predicted cleavage site that are different from *Rosavirus A* are in bold

### Phylogenetic analyses

**Fig 2:**
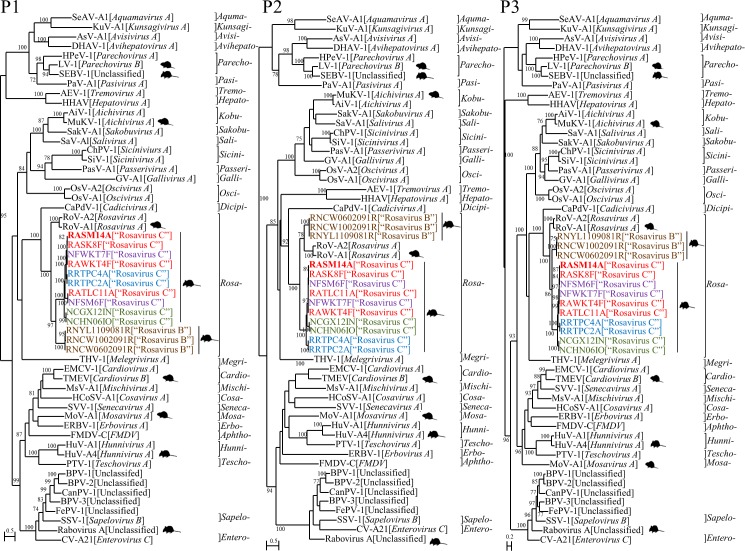
Phylogenetic analysis of the amino acid sequences of P1, P2 (excluding 2A) and P3 (excluding 3A) of the novel rodent picornavirus species with genomes completely sequenced. The strains identified in this study are shown in colors representing different host species of street rodents, Norway rats (strain number RNXXX) (brown), and wild rodents, Coxing's white-bellied rats (NCXXX) (green), roof rats (RRXXX) (blue), chestnut spiny rats (NFXXX) (purple) and Indochinese forest rats (RAXXX) (red) respectively. The strain RASM14A that was successfully isolated in 3T3 cells is shown in bold. The present and other known rodent picornaviruses are indicated by a pictorgram of rodent. The trees were constructed by maximum-likelihood method with bootstrap values calculated from 100 trees and only those >70% are shown. The scale bar indicates the estimated number of substitutions per 2 or 5 amino acids. BPV-1, bat picornavirus 1 (HQ595340); BPV-2, bat picornavirus 2 (HQ595342); BPV-3, bat picornavirus 3 (HQ595344); CanPV-1, canine picornavirus (JN831356); FePV-1, feline picornavirus (JN572117); FMDV-C, foot-and-mouth disease virus—type C (NC_002554); MuKV-1, murine kobuvirus 1 (JF755427).

Phylogenetic trees constructed using the aa sequences of P1, P2 (excluding 2A) and P3 (excluding 3A) of rosavirus B and C are shown in Fig 2 and the corresponding pairwise aa identities are shown in Table 2. 2A and 3A regions were excluded to avoid bias due to poor sequence alignment. In all three trees, the sequences from the present rodent picornaviruses formed two distinct clusters among known picornaviruses, being most closely related to *Rosavirus A* of the genus *Rosavirus*. However, their genomes shared only 56.3–59.8% nt identities with that of *Rosavirus A*. In the polyprotein, P1 and 2C/3C/3D regions, rosavirus B/C possessed only 57.3–57.4%/53.2–54%, 54.3–55%/51.1–52.5% and 65.9–66.1%/62.1–62.8% aa identities respectively to *Rosavirus A*. Moreover, rosavirus B and C shared only 58.9–60%, 57.7–59.4% and 67.6–68.3% aa identities respectively to each other in these regions. The predicted 2A protein of rosavirus B and C also showed low aa identities to that of rosavirus A, suggesting that they represent two novel picornavirus species, proposed to be named “Rosavirus B” and “Rosavirus C.”

### Genome features of rosavirus B and C

**Fig 3:**
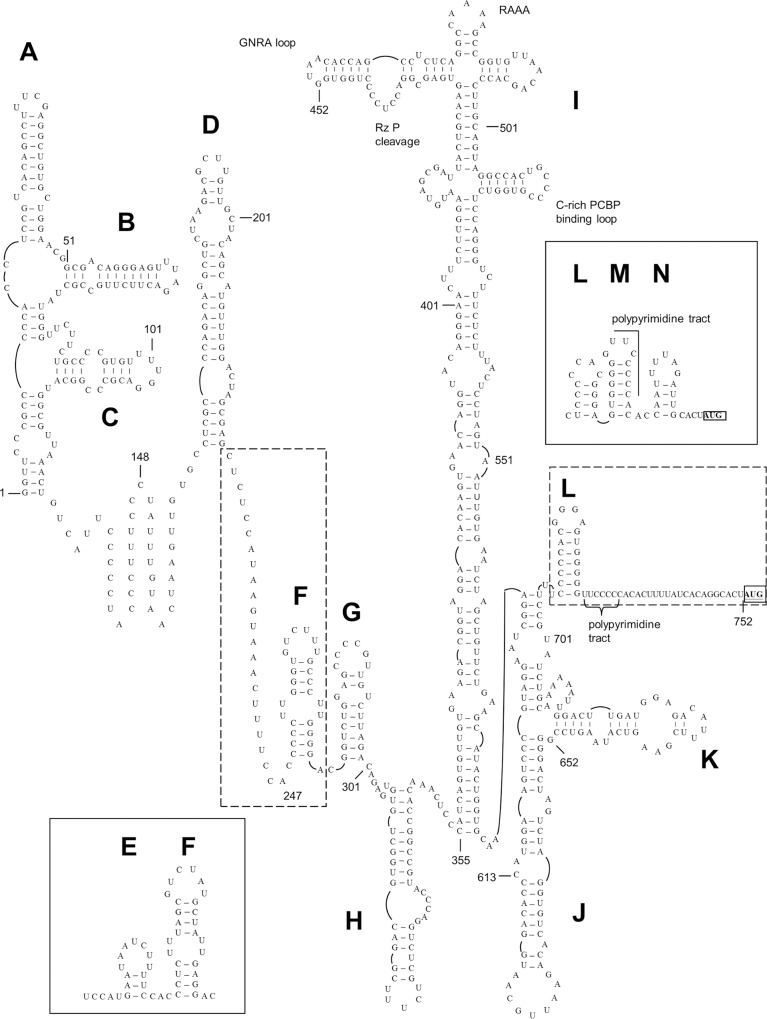
Predicted type II-like IRES structures of rosavirus C RRTPC2A. The stem-loop motifs/domains are labeled sequentially from A to N. Domains E/F and L/M/N were only observed in some strains of Rosavirus B and C. The AUG start codon was in bold and underlined.

Comparison of genome features of rosavirus B and C to those of rosavirus A is summarized in Table 4. The conserved sequence Yn-Xm-AUG is present in the 5'UTR of rosavirus B and C. While Y7-X19-AUG is found in rosavirus A, the number of Y (6 or 7) and X (19–21) varies among rosavirus B and C. The putative translation initiation sites were contained by an optimal Kozak context (RNNAUGG), with in frame AUG at position 471 to 753. The 5'UTR of many picornaviruses possesses an internal ribosomal entry site/segment (IRES) which is responsible for directing the initiation of translation in a cap-independent manner, and requires both canonical translation initiation and IRES *trans*-acting factors [3, 34]. Similar to rosavirus A [32], rosavirus B and C also contained a type II-like IRES with stem loops, major domains [19, 27, 35–40] and conserved motifs (Fig 3). However, domain E was only present in rosavirus C strains, RASK8F, RATLC11A, NFSM6F and RASM14A, and rosavirus B strain RNYL1109081R, but not other strains. The pyrimidine-rich region was located near 3' end of 5'UTR. One to three stem-loop structures were present upstream of the start codon and/or between the pyrimidine-rich region and start codon of the polyprotein [41, 42].

**Table 4:** Comparison of genomic and protein features of “Rosavirus B” and “Rosavirus C” to those of *Rosavirus A* and other representative genera in the *Picornaviridae* family.

Regions	Function, conserved motif or feature	Genera											
		*Cardio-*	*Dicipi-*	*Galli-*	*Kobu-* ^c^	*Megri-*	*Osci-*	*Parecho-*	*Passeri-*	*Sapelo-*	*Rosa*-	*Rosa-*	*Rosa*-
5’UTR	Pattern:Yn-Xm-AUG	Y9X18 (*Cardiovirus A*)	5’UTR, Y10X50; IGR, Y8X19 (*Cadiciviru A*)	Y10X32 (ChGV1)	Y7X11 (*Aichiviru A*)	No	Y6X19-34 (*Oscivirus A*)	Y7X20 (*Parechovirus A*)	Y9X19 (*Passeriviru A*)	Y16-17 X17-18 (*Sapelovirus B*)	Y7X18-19 (*Rosaviur A*)	Y7X19-20 (proposed “Rosavirus B”)	Y6-7X20-22 (proposed “Rosavirus C”)
	IRES[5, 72, 73]	Type II	Undefined	Type II-like	“Type V”^*^	Undefined	“Type V”	Type II	Undefined	Type IV	Type II	Type II	Type II
L	Protease [74, 75]	Y/N^a^	N	Y/N^a^	Y/N^a^	N	Y/N^a^	N	Y/N^a^	Y/N^a^	N	N	N
VP0	Cleaved into VP4 and VP2	Y	Y	N	N	N	N	N	N	Y	Y	Y	Y
	Myristylation site GXXX[ST] [43]:	Y	N	Y	Y	N	Y	N	Y	Y	Y	variable	N
VP1	Motif: [PS]ALXAXETG	N	N	N	N	N	N	N	N	Y	N	N	N
2A	Functions [44, 76]:	NPGP	Unknown^b^	H-box/NC	H-box/NC	H-box/NC	Unknown^b^	H-box/NC in HPeV and LV; NPGP in LV	H-box/NC	Chymotrypsin-like protease or unknown^b^	H-box/NC	H-box/NC	H-box/NC
2C	NTPase motif GXXGXGKS [46]:	Y	Y	Y	Y	Y	Y	Y	Y	Y	Y	Y	Y
	Helicase DDLXQ [47]	Y	Y	DDIGQ	DD[LI]GQ	DDFCQ	Y	DD[LA]GQ	DDVGQ	Y	Y	Y	Y
3C^pro^	Catalytic triad H-D/E-C [49]:	H-D-C	H-E-C	H-E-C	H-E-C	H-D-C	H-E-C	H-D-C	H-E-C	H-E-C	H-D-C	H-D-C	H-D-C
	RNA-binding domain motif KFRDI [50]	N	N	N	N	N	N	N	N	Y	N	N	N
	GXCG, GXH motif [49]	Y	Y	Y	Y	Y	Y	Y	Y	Y	Y	Y	Y
3D^pol^	Motif: KDE[LI]R [51]	Y	Y	Y	Y	Y	Y	Y	Y	Y	Y	Y	Y
	Motif: GG[LMN]PSG [51]	Y	GAMPSG	Y	Y	Y	GGMPS[GR]	Y	Y	Y	GAMPSG	GAMPSG	GAMPSG
	Motif: YGDD [51]	Y	Y	Y	Y	Y	Y	Y	Y	Y	Y	Y	Y
	Motif: FLKR[51]	Y	Y	Y	Y	Y	Y	Y	Y	Y	Y	Y	Y

Y, present; N, absent.

^a^Y/N, Presence of L but L is not protease.

^b^2A of *Cadiciviurs A*, *Oscivirus A* and *Sapelovirus A* do not contain the characteristic catalytic amino acid residues with chymotrypsin-like proteolytic activity, the NPGP motif or the H-box/NC motif.

^c^Porcine kobuvirus SUN-1-HUN was predicted to have type IV IRES.

The predicted “VP0” of rosavirus B and C are probably cleaved into VP4 and VP2 based on sequence alignment [32]. In contrast to rosavirus A of which the VP4 possessed the myristylation site, GXXX[ST], involved in capsid assembly or virus entry [43], such myristylation site is absent in rosavirus C and variably present in rosavirus B (present in strains RNCW0602091R and RNCW1002091R but absent in strain RNYL1109081R). The predicted 2A of rosavirus B and C exhibited ≤45.1% aa identities to that of rosavirus A, possessed the conserved H-box/NC involved in cell proliferation control, but not Asn-Pro-Gly-Pro (NPGP) motifs [44, 45]. Their predicted 2C possessed GXXGXGKS motif for NTP-binding [46] and DDLXQ motif for putative helicase activity [47]. Their predicted 3C^pro^ contained the catalytic triad H-D-C [48], conserved GXCG motif in the protease active site and GXH motif [49, 50]. Their predicted 3D^pol^ contained conserved KDE[LI]R, GG[LMN]PSG, YGDD and FLKR motifs [51], although the second Gly was replaced by Ala in GG[LMN]PSG.

Although rosavirus C were detected in five different rodent species from Hong Kong, Hunan and Guangxi, no major distinct genome features were identified between strains from different rodent species or geographical locations. Yet, the two strains from Coxing’s white-bellied rat from Hunan (NCHN06IO) and Guangxi (NCGX12IN), were always clustered together in the P1, P2 and P3 trees, suggesting that geographically distinct strains may be genetically closely related (Fig 2). These two strains from mainland China possessed a total of 432 unique nucleotide substitutions over the entire genomes compared to the other eight rosavirus C strains from Hong Kong.

Viral sequences belonging to “Rosavirus B” were only detected from the street rodent species, Norway rat (*Rattus norvegicus*), whereas sequences belonging to “Rosavirus C” were detected from five different wild rodent species, greater bandicoot rat (*Bandicota indica*), chestnut spiny rat (*Niviventer fulvescens*), roof rat (*Rattus rattus*) and Indochinese forest rat (*Rattus andamanensis*) from Hong Kong, and

### Estimation of synonymous and non-synonymous substitution rates

Using available rosavirus B and C genome sequences for analysis, the Ka/Ks ratios for various coding regions were estimated (Table 5). The Ka/Ks ratios for most coding regions were low, supporting purifying selection.

**Table 5:** Estimation of nonsynonymous and synonymous substitution rates in the genomes of “Rosavirus B” and “Rosavirus C.”

	“Rosavirus B” (n = 3)			“Rosavirus C” (n = 10)		
Gene	Ka	Ks	Ka/Ks	Ka	Ks	Ka/Ks
P1						
VP4	0.011	0.045	0.244	0.002	0.164	0.012
VP2	0.062	0.872	0.071	0.087	0.921	0.094
VP3	0.006	0.674	0.009	0.074	1.129	0.066
VP1	0.007	0.501	0.014	0.111	1.068	0.104
P2						
2A	0.027	0.417	0.065	0.116	1.023	0.113
2B	0.005	0.339	0.015	0.088	0.822	0.107
2C	0.005	0.414	0.012	0.047	1.283	0.037
P3						
3A	0.012	0.36	0.033	0.106	1.045	0.101
3B	0.011	0.376	0.029	0.131	0.575	0.228
3C	0.006	0.213	0.028	0.051	0.928	0.055
3D	0.003	0.294	0.010	0.054	1.059	0.051

### Isolation of rosavirus C in 3T3 cells

**Fig 4:**
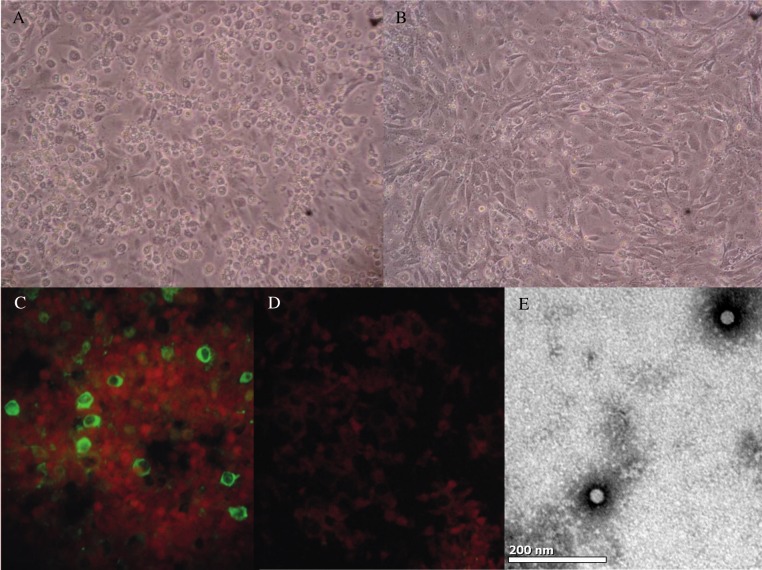
**(A) 3T3 cells infected with rosavirus C RASM14A showing cytopathic effects with rounded and refractive cells rapidly detaching from the monolayer at day 5 after incubation, compared to (B) uninfected cells.** (C) Immunofluorescence staining of 3T3 cells infected with rosavirus C RASM14A, compared to (D) uninfected cells using guinea pig antiserum against VP1. (E) Negative contrast electron microscopy of ultracentrifuged deposit of 3T3 cells culture-grown rosavirus C RASM14A, showing non-enveloped picornaviral particles of around 25–30 nm in diameter, bar = 50 nm.

Of the various cell lines inoculated with the 11 rodent samples positive for rosavirus B (three samples) or rosavirus C (eight samples), viral replication was detected by RT-PCR in the lysates of 3T3 cells infected by rosavirus C strain RASM14A, with viral load of 4.5×10^8^ copies/ml (3.2 × 10^3^ TCID_50_) at day 7. Cytopathic effect (CPE), mainly in the form of rounded and refractile cells rapidly detaching from the monolayer, was also observed in infected 3T3 cells five days after inoculation, which showed viral VP1 expression by immunofluorescence in 40% of cells (Fig 4). Electron microscopy of ultracentrifuged cell culture extracts from infected 3T3 cells showed the presence of non-enveloped viral particles of around 25–30 nm in diameter compatible with those described for members of the family of *Picornaviridae* (Fig 4).

### Seroprevalence of rosavirus B and C

**Fig 5:**
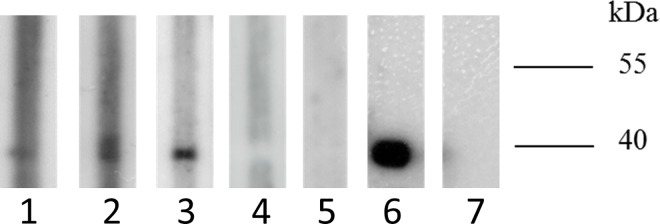
Western blot analysis for detection of antibodies against purified His6-tagged recombinant VP1 proteins of rosavirus B strain RNCW1002091R from a Norway rat and rosavirus C strains, RASK8F from an Indochinese forest rat and NCGX12IN from a Coxing's white-bellied rat (~40-kDa) in rodent serum samples. Representatives of results are shown. Lanes: 1, Norway rat serum sample positive for antibody against rosavirus B strain RNCW1002091R VP1 protein; 2, Indochinese forest rat serum sample positive for antibody against rosavirus C strain RASK8F VP1 protein; 3, Coxing's white-bellied rat serum sample positive for antibody against rosavirus C strain NCGX12IN VP1 protein; 4, rosavirus B-VP1 antibody-positive Norway rat serum sample against rosavirus C strain NCGX12IN VP1 protein; 5, rosavirus C-VP1 antibody-positive Coxing's white-bellied rat serum sample against rosavirus B strain RNCW1002091R VP1 protein; 6, positive control (anti-His antibody); 7, negative control.

To determine the seroprevalence of rosavirus B and C among different rodent species, western blot analysis was performed on available rodent serum samples to test for specific antibodies against rosavirus B or C recombinant VP1 protein. The purity of the recombinant VP1 proteins was confirmed by the dominant band observed at the predicted size of 40 kDa upon SDS polyacrylamide gel electrophoresis. Anti-rosavirus B antibodies were detected in two (3.3%) of 61 Norway rats from Hong Kong whereas anti-rosavirus C antibodies were detected in three (4.3%) of 70 Indochinese forest rats from Hong Kong and three (7.5%) of 40 Coxing's white-bellied rats from Hunan Province. However, the antibodies from Norway rats against rosavirus B were likely of low levels, as reflected by the relatively weak band observed (Table 1 and Fig 5). Using sera with anti-rosavirus B antibodies against rosavirus C recombinant VP1 protein and sera with anti-rosavirus C antibodies against rosavirus B recombinant VP1 protein, no cross reactivities were observed between the two proteins. Neutralization assays showed that five of the six rats with anti-rosavirus C antibodies by western blot analyses were positive for neutralizing antibodies against rosavirus C RASM14A with titer 1:10 to 1:40.

### Pathogenicity of rosavirus C in challenged mice

**Fig 6:**
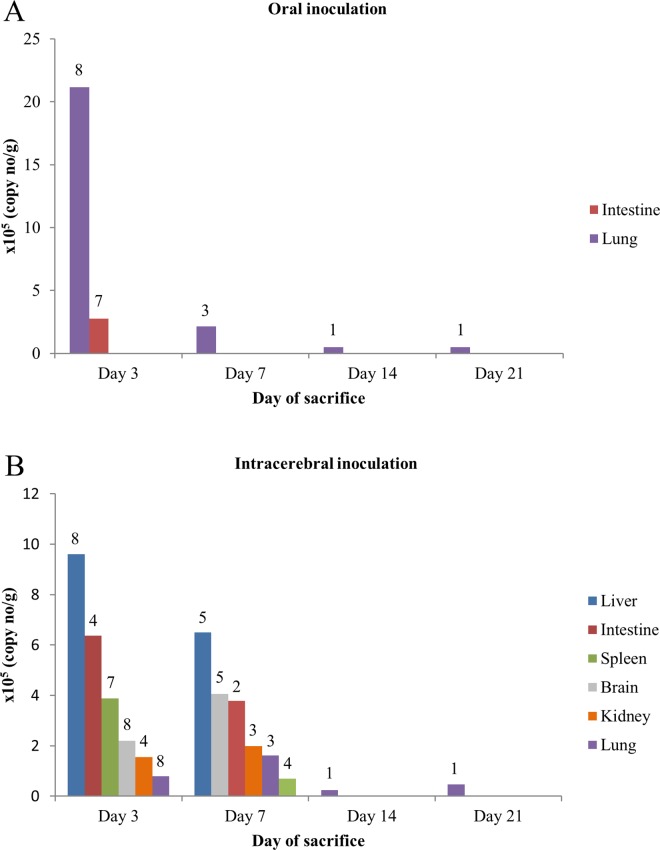
**Viral loads by qRT-PCR of RT-PCR-positive tissues from sacrificed mice challenged with rosavirus C RASM14A by oral (A) and intracerebral (B) inoculation.** Numbers above the bars indicate the number of tested mice with RT-PCR-positive tissues.

We attempted to study the pathogenicity in mice challenged with rosavirus C RASM14A isolated from infected 3T3 cells. To mimick the fecal-oral route of transmission typical of many picornavirus infections, oral inoculation of rosavirus C RASM14A was performed on 21 four-day-old suckling mice. One of the suckling mice was eaten by its mother on day one post-challenge. All the remaining 20 suckling mice survived after viral challenge till sacrifice, but some mice exhibit transient roughening of hair two to three days after challenge. Among the nine mice sacrificed on day 3 post-challenge, rosavirus C RASM14A was detected in the intestine and lung of all nine mice, kidney of one mouse, and spleen and liver of three and four mice respectively by RT-PCR. Among the five mice sacrificed on day 7 post-challenge, rosavirus C RASM14A was detected in the intestine of all five mice, liver and lung of four mice, and spleen and kidney of two and one mice respectively by RT-PCR. Among the three mice sacrificed on day 14 post-challenge, rosavirus C RASM14A was detected in the lung of one mouse by RT-PCR. Among the three mice sacrificed on day 21 post-challenge, rosavirus C RASM14A was detected in the lung of one mouse by RT-PCR. Anti-rosavirus C VP1 antibody was detected in none of the mice sacrificed on day 3 and 14, two of the five mice sacrificed on day 7, and one of the three mice sacrificed on day 21 by Western blot assay (Table 6). qRT-PCR of tissues positive by RT-PCR showed high levels of mean viral RNA copies in lung (2.1 ×10^6^ copies/g) and intestine (2.8 ×10^5^ copies/g) tissues of mice sacrificed on day 3 (Fig 6).

**Table 6:** RT-PCR and western blot analysis of mice challenged with rosavirus C RASM14A.

Route of infection	Day at which mice were sacrificed (total no. of mice)	No. of tissues/sera (%) from sacrificed mice tested positive for rosavirus C RASM14A by RT-PCR/western blot analysis						
		Brain	Intestine	Spleen	Kidney	Liver	Lung	Serum antibody
Oral inoculation	Day 3 (9)	0 (0%)	9 (100%)	3 (33.3%)	1 (11.1%)	4 (44.4%)	9 (100%)	0 (0%)
	Day 7 (5)	0 (0%)	5 (100%)	2 (40%)	1 (20%)	4 (80%)	4 (80%)	2 (40%)
	Day 14 (3)	-	0 (0%)	0 (0%)	0 (0%)	0 (0%)	1 (33.3%)	0 (0%)
	Day 21 (3)	-	0 (0%)	0 (0%)	0 (0%)	0 (0%)	1 (33.3%)	1 (33.3%)
Intracerebral inoculation	Day 3 (9)	8 (88.9%)	4 (44.4%)	8 (88.9%)	4 (44.4%)	8 (88.9%)	8 (88.9%)	0 (0%)
	Day 7 (5)	5 (100%)	2 (40%)	4 (80%)	4 (80%)	5 (100%)	3 (60%)	4 (80%)
	Day 14 (3)	1 (33.3%)	1 (33.3%)	0 (0%)	0 (0%)	1 (33.3%)	1 (33.3%)	2 (66.7%)
	Day 21 (3)	0 (0%)	0 (0%)	0 (0%)	0 (0%)	0 (0%)	1 (33.3%)	2 (66.7%)

Since some picornaviruses have been associated with neurovirulence, intracerebral inoculation was also performed on another group of 21 one-day old suckling mice. One of the suckling mice was eaten by its mother on day one post-challenge. All the remaining 20 suckling mice survived after viral challenge till sacrifice. Among the nine mice sacrificed on day 3 post-challenge, rosavirus C RASM14A was detected in the lung, liver, brain and spleen of eight mice, and intestine and kidney of four mice by RT-PCR. Among the five mice sacrificed on day 7 post-challenge, rosavirus C RASM14A was detected in the lung of three mice, brain and liver of five mice, intestine of two mice, and spleen and kidney of four mice by RT-PCR. Among the three mice sacrificed on day 14 post-challenge, rosavirus C RASM14A was detected in the brain, intestine, liver and lung of one mouse by RT-PCR. Among the three mice sacrificed on day 21 post-challenge, rosavirus C RASM14A was detected in the lung of one mouse by RT-PCR. Anti-rosavirus C VP1 antibody was detected in none of mice sacrificed on day 3, four of the five mice sacrificed on day 7, and two of three mice sacrificed on day 14 and 21 by Western blot assay (Table 6). qRT-PCR of tissues positive by RT-PCR showed high levels of mean viral RNA copies in various tissues (0.8 to 9.6 ×10^5^ copies/g) of mice sacrificed on day 3 (Fig 6).

**Fig 7:**
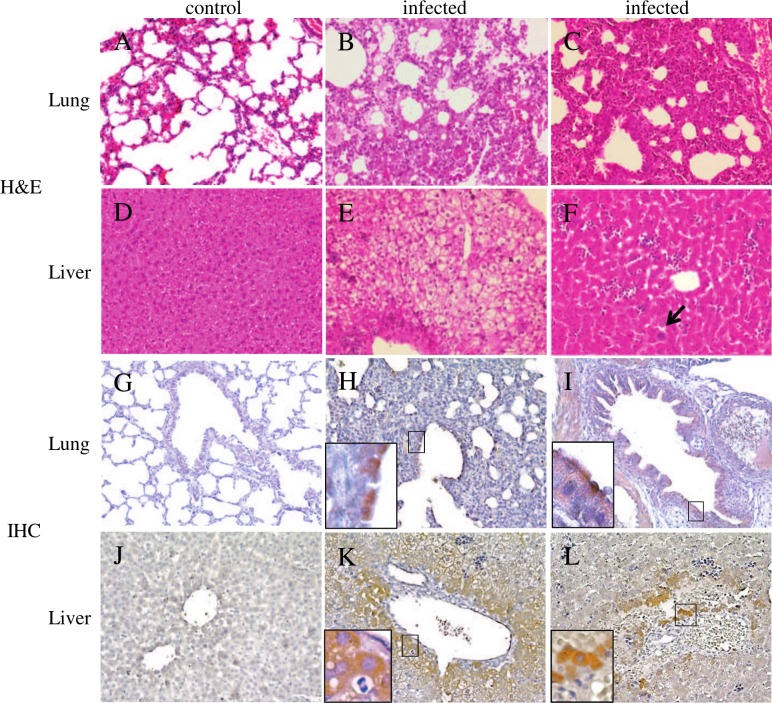
Histological changes and immunohistochemical staining of mice lung and liver tissues on day 3 after oral or intracerebral inoculation of rosavirus C RASM14A with micrographs at magnification 200×. A-C, Representative images of H&E staining of lung sections of control mouse (A) and infected mice showing alveolar fluid exudation (B) and increased cellularity, interstitial infiltration and alveolar wall thickening (C); D-F, Representative images of H&E staining of liver sections of control mouse (D) and infected mice showing hepatocyte degeneration (E) and lymphocytic/monocytic inflammatory infiltration and giant cell formation (arrow) (F); G-L, Immunohistochemical staining of lung and liver sections using guinea pig anti-serum against rosavirus C VP1 protein antibody; G and J are lung and liver sections from control mice with inoculation of uninfected cell culture medium respectively; H and I, lung sections of infected mice showing bronchiolar and bronchial epithelial cells positively stained with rosavirus C VP1 protein in brown colour (insert, magnification 400×); K and L, liver sections of infected mice showing positive staining of rosavirus C VP1 protein in hepatocytes with brown colour (insert, magnification 400×).

Histological examination of various organs revealed alveolar fluid exudation, interstitial infiltration, alveolar fluid exudate and wall thickening in lung sections of mice sacrificed on day 3 after oral or intracerebral inoculation. Moreover, hepatocyte degeneration and lymphocytic/monocytic inflammatory infiltrates with giant cell formation were observed in liver sections of mice sacrificed on day 3 after oral or intracerebral inoculation (Fig 7). Immunohistochemical staining with guinea pig anti-serum against rosavirus C VP1 protein antibody revealed viral antigen expression in bronchiolar and bronchial epithelial cells in lung sections, and hepatocytes in liver sections (Fig 7).

## Discussion

We report the discovery of two novel rodent picornaviruses, rosavirus B and C, from six rodent species in southern China. Though being phylogenetically most closely related to *Rosavirus A*, “Rosavirus B” and “Rosavirus C” should represent two novel species distinct from *Rosavirus A* under the genus *Rosavirus*, according to the criteria for International Committee on Taxonomy of Viruses species demarcation for different members of *Picornaviridae* [52]. Rosavirus B and C also exhibited different genome features when compared to rosavirus A. Notably, the absence of myristylation site in VP4 of rosavirus C and its varying presence in rosavirus B is intriguing. The VP4 myristylation site has been shown to play a role in localization of the capsid protein for cellular entry and permeability [43, 53]. Its absence in some rosavirus strains suggests that rosaviruses may utilize alternative strategies for capsid localization on cellular targets. In addition, the variations in Yn-Xm-AUG sequence in 5’UTR may also suggest different translational dynamics among rosaviruses. Besides phylogenetic and genomic evidence, the two viruses infect different host, with “Rosavirus B” infecting Norway rats (a street rat) and “Rosavirus C” infecting various wild rats, as supported by positive specific and/or neutralizing antibodies in the respective animals. Further studies are required to better understand the epidemiology of these novel rosaviruses in other rodent population.

Our findings support the potential of rosaviruses for interspecies transmission, and extend our knowledge on the diversity and host range of picornaviruses in rodents. Picornaviruses belonging to *Cardiovirus*, *Hunnivirus*, *Kobuvirus*, *Mosavirus*, *Parechovirus* and *Rosavirus* are known to infect different rodents [32, 33, 54]. Rosavirus A was first discovered in a wild canyon mouse (*Peromyscus crinintus*) in California [32]. Subsequently, a variant, rosavirus 2, was detected in the fecal specimens of children in the Gambia [26], which prompted further studies to investigate potential transmission of rosavirus from rodents to humans. In this study, the observed low Ka/Ks ratios in various coding regions supported that Norway rats (street rats) in Hong Kong, and wild rats across Hong Kong and mainland China, are natural reservoirs of rosavirus B and C respectively. In particular, Coxing’s white-bellied rats appeared to be an important host for rosavirus C. The relatively low seroprevalence of rosavirus C, as compared to RT-PCR detection rate, among tested Coxing’s white-bellied rats (7.5%) may be due to delayed antibody response during acute infection when the animals were still shedding viruses. Together with the ability of rosavirus C in infecting house mouse (*Mus musculus*), our findings provided evidence for the interspecies transmission potential of rosavirus C among different rodent species. This is in line with the ability of bat picornaviruses group 1 to 3 in infecting bats of different genera or species [29]. Encephalomyocarditis virus (EMCV), of which swine is the major reservoir, can also infect different animals including rodents, elephants, boars, macaques and humans [55]. Further investigations are warranted to elucidate the ability of rosaviruses to cross species barrier and emerge in other animals or human.

The present results suggest that rosaviruses can be pathogenic to their hosts. Although rosavirus A has been detected in rodents and human previously [26, 32], no virus isolate was available for pathogenicity studies. A few picornaviruses, such as EMCV (cardiovirus A) and Theilovirus (cardiovirus B) under *Cardiovirus* and Ljungan virus (LV) (parechovirus B) under *Parechovirus*, were also known to cause systemic infections in infected rodents. In particular, Theiler's murine encephalomyelitis virus (TMEV), which primarily causes asymptomatic enteric infections in mice, has been intensively studied because of its ability to cause myocarditis, type 1 diabetes and acute or persistent demyelinating infections mimicking multiple sclerosis [35, 56]. On the other hand, rodents experimentally infected with EMCV may develop type 1 diabetes mellitus, encephalomyelitis, myocarditis, orchitis and sialodacryoadenitis [57]. Interestingly, LV, which may cause type 1 diabetes and fetal deaths in infected rodents, has been recently found in human intrauterine fetal death and sudden infant death syndrome [58–61]. However, the pathogenicity of other rodent picornaviruses, such as rosavirus A1, mosavirus A1, murine kobuvirus 1, Norway rat hunnivirus and rabovirus A, was less clear [32, 33]. The ability of rosavirus C in causing multisystemic infection in mice with high viral loads in infected organs suggested that rosaviruses may cause severe infections in their host. Further experimental studies using other rosaviruses and rodent species may help to better understand the pathogenicity of members of the genus *Rosavirus* in different rodents and human.

Rodents are the largest order of mammals on earth, accounting for 43% of the approximately 4,800 living mammalian species. They are widely distributed, being found in all habitats except the oceans. The order, *Rodentia*, with around 2050 species under 28 families, is further classified into five suborders: *Anomaluromorpha*, *Castorimorpha*, *Hystricomorpha*, *Myomorpha and Sciuromorpha*. [62, 63]. Viruses of at least 22 families, including *Adenoviridae*, *Arenaviridae*, *Arteriviridae*, *Astroviridae*, *Bunyaviridae*, *Caliciviridae*, *Circoviridae*, *Coronaviridae*, *Flaviviridae*, *Hepadnaviridae*, *Hepeviridae*, *Herpesviridae*, *Papillomaviridae*, *Paramyxoviridae*, *Parvoviridae*, *Picobirnaviridae*, *Polyomaviridae*, *Reoviridae*, *Rhabdoviridae*, *Togaviridae*, *Picornaviridae* and *Poxviridae*, are known to infect rodents [64, 65]. Rodent pathogens may infect human either by direct contact such as bites and inhalation of aerosolized animal excreta, or indirectly through vectors such as ticks and fleas. Urban rodents may pose particular risk to human health, as in the case of Hantavirus and lymphocytic choriomeningitis virus infections. More epidemiological studies should be performed to explore the diversity of rodent picornaviruses and their potential risks to human.

## Materials and Methods

### Collection of rodent samples

A total of 1232 wild and street rodents, belonging to eight different species, were captured from various locations in both rural and urban areas of Hong Kong, Hunan Province and Guangxi Province of China over a five-year period (September 2008 to August 2013) (Table 1). Samples from Hong Kong were provided by the Agriculture, Fisheries and Conservation Department (AFCD) and Food, Environment and Hygiene Department (FEHD), the government of the Hong Kong Special Administrative region (HKSAR), as part of a surveillance program on local rodents. All rodents were individually trapped and samples were collected from each rodent using procedures described previously [11, 66]. To prevent cross contamination, collection of samples were performed using disposable swabs with protective gloves changed for each rodent. Wild rodents in rural areas of Hong Kong were released back to nature after sample collection. Samples from street rodents in urban areas of Hong Kong and rodents from China were collected immediately after euthanasia as routine policies for disposal of captured rodents. All samples were placed in viral transport medium (Earle’s balanced salt solution, 0.09% glucose, 0.03% sodium bicarbonate, 0.45% bovine serum albumin, 50 mg/ml amikacin, 50 mg/ml vancomycin, 40 U/ml nystatin) to inhibit bacterial and fungal overgrowth, and stored at -80°C before RNA extraction.

### RNA extraction

Viral RNA was directly extracted from the respiratory and alimentary samples in viral transport medium using Viral RNA mini kit (QIAgen, Hilden, Germany). The RNA was eluted in 60 μl of RNase-free water and was used as the template for RT-PCR.

### RT-PCR of 3D^pol^ gene of picornaviruses using conserved primers and DNA sequencing

Initial picornavirus screening was performed by amplifying a 159-bp fragment of the 3D^pol^ gene of picornaviruses by RT-PCR using conserved primers (5’-GGCGGYTNGAYGGYGCSATGCCGT-3’ and 5’-CCGACCARCACRTCRTCRCCRTA-3’) and previously described protocols [6, 24, 29]. The primers were designed by multiple alignment of the nucleotide sequences of the 3D^pol^ genes of all known picornaviruses, based on the conserved 3D^pol^ motifs, GG[LMN]PSG and YGDD. All samples positive by RT-PCR were confirmed by sequencing. Briefly, reverse transcription was performed using the SuperScript III kit (Invitrogen, San Diego, CA, USA) and the reaction mixture (10 μl) contained RNA, first-strand buffer (50 mM Tris-HCl pH 8.3, 75 mM KCl, 3 mM MgCl_2_), 5 mM DTT, 50 ng random hexamers, 500 μM of each dNTPs and 100 U Superscript III reverse transcriptase. The mixtures were incubated at 25°C for 5 min, followed by 50°C for 60 min and 70°C for 15 min. The PCR mixture (25 μl) contained cDNA, PCR buffer (10 mM Tris-HCl pH 8.3, 50 mM KCl, 2 mM MgCl_2_ and 0.01% gelatin), 200 μM of each dNTPs and 1.0 U *Taq* polymerase (Applied Biosystem, Foster City, CA, USA). The mixtures were amplified in 40 cycles of 94°C for 1 min, 50°C for 1 min and 72°C for 1 min and a final extension at 72°C for 10 min in an automated thermal cycler (Applied Biosystem, Foster City, CA, USA). Standard precautions were taken to avoid PCR contamination and no false-positive was observed in negative controls.

All PCR products were gel-purified using the QIAquick gel extraction kit (QIAgen, Hilden, Germany). Both strands of the PCR products were sequenced twice with an ABI Prism 3130xl DNA Analyzer (Applied Biosystems, Foster City, CA, USA), using the two PCR primers. The sequences of the PCR products were compared with known sequences of the 3D^pol^ genes of picornaviruses in the GenBank database.

### RT-PCR of 3D^pol^ gene of novel rodent picornaviruses using specific primers and DNA sequencing

As initial RT-PCR of the 3D^pol^ gene revealed at least two potential novel picornavirus species in 18 respiratory and 24 alimentary tract samples, all the 2450 respiratory and alimentary tract samples were re-tested using specific RT-PCR assays to enhance the sensitivities for detection of these novel picornaviruses. Primers were designed by multiple alignment of the 3D^pol^ gene sequences obtained during genome sequencing from the initial positive samples. The PCR assays were targeted to a 253 bp (5’- ATGCTCCTGTTCTCATGCTTTT -3’ and 5’- GAAAATCTGGGTCAGGGGTGAA -3’) fragment and a 243-bp (5’- TGTTCTCTTGYTTYTCCCAGAT -3’ and 5’- AAYTGCGGGTCYGGDGTGAA -3’) fragment of the 3D^pol^ gene of the potential novel picornaviruses. The components of the PCR mixtures and the cycling conditions were the same as those described above. Purification of the PCR products and DNA sequencing were performed as described above, using the corresponding PCR primers. The sequences of the PCR products were compared with known sequences of the 3D^pol^ genes of picornaviruses in the GenBank database.

### Real-time quantitative RT-PCR (qRT-PCR)

Real-time RT-qPCR was performed on samples positive for the novel picornaviruses by RT-PCR as described previously [1, 29]. Briefly, specific primers targeting a 144-bp (5’-TGTCAGATGGTGTCAACAGTCAAA-3’ and 5’-TCATGGCGCACTTTCACATT-3’), a 137-bp (5’-ACAAATCTACAGCCAAATTCCAAA-3’ and 5’-GTAGGGTATGCCTTTCTGGTCAA-3’) and a 112-bp (5’-CAGCCAAATTCCAAATTCAGAT-3’ and 5’-CCAGATCAGCCATGTTTGGAA-3’) fragment of the 2C genes were used for RT-qPCR by Thermal Cyler FastStart DNA Master SYBR Green I Mix reagent kit (Roche). cDNA was amplified by Thermal Cycler 7900HT (Applied Biosystems) with 20-μl reaction mixtures containing FastStart DNA Master SYBR Green I Mix reagent kit (Roche). A plasmid containing the target sequence was used for generating the standard curves.

### Genome sequencing and analysis

Thirteen genomes of the two novel picornavirus species were amplified and sequenced, with RNA directly extracted from respiratory or alimentary samples as templates [6, 24, 29]. RNA was converted to cDNA by a combined sequence-specific-priming, random-priming and oligo (dT) priming strategy. As initial results showed that the two novel picornaviruses are distantly related to known picornaviruses, the cDNAs of three initial strains were amplified by 5’-rapid amplification of cDNA ends (RACE) using the SMARTer RACE cDNA Amplification Kit (Clontech, USA). The first strand cDNA for the 5’ sequence of the genome was constructed with specific primers designed according to results of the first and subsequent rounds of sequencing and SMARTer II A Oligonucleotide by SMARTScribe Reverse Transcriptase. The 3’ sequence of the genome is completed by specific primers designed for the 3’ end from the results of the first and subsequent rounds of sequencing and oligo (dT) primer. Sequences were assembled to produce final sequences of the viral genomes. The genomes of the remaining strains were amplified and sequenced by the specific primers designed from the initial three genomes and the 5’ ends of the viral genomes were confirmed by RACE using the SMARTer RACE cDNA Amplification Kit (Clontech, USA).

The nucleotide (nt) sequences of the genomes and the deduced amino acid (aa) sequences of the open reading frames (ORFs) were compared to those of known picornaviruses. Phylogenetic tree construction was performed using maximum-likelihood methods from PhyML 3.0 program. Secondary structure prediction in the 5’UTR was performed using RNAfold [67] and the IRES elements were determined based on sequence alignment with EMCV as described previously [28, 41].

### Estimation of synonymous and non-synonymous substitution rates

To estimate the selective pressure in driving viral evolution among different regions of the genomes, the number of synonymous substitutions per synonymous site, Ks, and the number of non-synonymous substitutions per non-synonymous site, Ka, for each coding region between different strains of rosavirus B and C were calculated using the Nei-Gojobori method (Jukes-Cantor) in MEGA 5.0 as described previously [68].

### Western blot analysis

Since the VP1 is the largest and most surface-exposed protein which contains most of the motifs important for interaction with neutralizing antibodies in picornaviruses, (His)_6_-tagged recombinant VP1 proteins of rosavirus B strain RNCW1002091R from a Norway rat and rosavirus C strains, RASK8F from an Indochinese forest rat and NCGX12IN from a Coxing's white-bellied rat, were cloned as described previously [28, 30]. Briefly, the VP1 gene was amplified and cloned into the NheI site of expression vector pET-28b(+) (Novagen, Madison, WI, USA) in frame and downstream of the series of six histidine residues. The (His)_6_-tagged recombinant VP1 polypeptide was expressed and purified using the Ni^2+^-loaded HiTrap Chelating System (GE Healthcare, Buckinghamshire, UK) according to manufacturer’s instructions. Western blot analysis was carried out using available rodent sera using the purified recombinant VP1 protein as described previously [30]. Briefly, the purified (His)_6_-tagged recombinant VP1 protein was loaded into each well of a sodium dodecyl sulfate (SDS)–10% polyacrylamide gel and subsequently electroblotted onto a nitrocellulose membrane (Bio-Rad, Hercules, CA, USA). The blot was cut into strips and the strips were incubated separately with serial dilutions of sera collected from different rodent species with for IgG detection. Antigen-antibody interaction was detected with horse radish peroxidase-conjugated secondary antibodies and ECL fluorescence system (GE Healthcare, Buckinghamshire, UK).

### Viral cultures

Eleven samples tested positive for the novel picornaviruses were subject to virus isolation in various cell lines including Vero E6 (African green monkey kidney; ATCC CRL-1586), CrFK (Crandell feline kidney; ATCC CCL-94), in-house HFL (human embryonic lung fibroblast), 3T3 (mouse embryonic fibroblast, ATCC CCL-92) cells, RD (human embryo rhabdomyosarcoma; ATCC CCL-136), RK3E (rat kidney; ATCC CRL-1895) and TCMK1 (mouse kidney; ATCC CCL-139) cells as described previously [28, 69]. Briefly, after centrifugation, samples were diluted five folds with viral transport medium and filtered. Filtrates were inoculated to Minimum Essential Media (MEM) and the mixtures were added to 24-well tissue culture plates by adsorption inoculation. After 1 h of adsorption, excess inoculum was discarded, and the wells were washed twice with phosphate buffered saline and replaced by serum-free MEM. Cultures were inspected daily by inverted microscopy for CPE. Subculturing to fresh cell line was performed from time to time even if there was no CPE and culture lysates were collected for RT-PCR for monitoring viral replication. Immunostaining and electron microscopy were performed on samples that were RT-PCR positive.

### Electron microscopy

3T3 cells successfully infected by rosavirus C RASM14A were subject to negative contrast electron microscopy as described previously [69, 70]. Tissue culture cell extracts infected with rosavirus C RASM14A were centrifuged at 19 000 g at 4°C, after which the pellet was resuspended in phosphate-buffered saline and stained with 2% phosphotungstic acid. Samples were examined with a Philips EM208s electron microscope.

### Neutralization assays

Neutralization assays for rosavirus C RASM14A were carried out as described previously with modifications [11]. Briefly, rodent sera serially diluted from 1:10 to 1:80 were mixed with 100 TCID_50_ of rosavirus C RASM14A. After incubation for 2 h at 37°C, the mixture was inoculated in duplicates onto 96-well plates of 3T3 cell cultures. Results were recorded after 3 days of incubation at 37°C.

### Mice challenge experiments

Virus stock used to inoculate mice was obtained from at least the 18^th^ passage of rosavirus C RASM14A in 3T3 cells. Groups of 20 suckling balb/c mice were infected orally (4-day-old) and intracerebrally (1-day-old) as described previously [71]. Approximately 200μl (500 TCID_50_) of virus suspensions was applied orally and 30μl (100 TCID_50_) intracerebrally. Two mice challenged with culture media from uninfected cells were included as negative controls in both groups. Mice were monitored daily for signs of disease. Nine/ten, five, three and three mice were sacrificed at day 3, 7, 14 and 21 respectively. After euthanasia, necropsies of mice were performed to obtain the following tissues: intestine, spleen, kidney, liver, lung and brain. Blood was collected for antibody tests by western blot analysis as described above.

### Histopathological and immunohistochemical studies

To perform immunhistochemical staining on infected cell lines and rodent tissues, guinea pig antiserum against the VP1 protein of rosavirus C was produced by subcutaneously injecting 100 μg purified recombinant rosavirus C VP1 protein to three guinea pigs, using an equal volume of complete Freund’s adjuvant (Sigma) as described previously [28]. Incomplete Freund’s adjuvant (Sigma) was used in subsequent immunizations. Three inoculations at once every two weeks per guinea pig were administered. Two weeks after the last immunization, 1 ml of blood was taken via the lateral saphenous vein of the guinea pigs to obtain the sera.

To examine the histopathology and viral replication of rosavirus C RASM14A in tissues of challenged mice, necropsy organs of the mice were subject to both viral RNA detection by RT-PCR and immunohistological studies as described previously [28]. Tissues for histological examination were fixed in 10% neutral-buffered formalin, embedded in paraffin, and stained with hematoxylin and eosin (H&E). Histopathological changes were observed using Nikon 80*i* microscope and imaging system. Infected cell lines and tissues from challenged mice that were tested positive for rosavirus C RASM14A by RT-PCR were subject to viral load studies and immunohistochemical staining for viral VP1 protein as described previously [28, 69]. Tissue sections were deparaffinized and rehydrated, followed by blocking endogenous peroxidase with 0.3% H_2_O_2_ for 25 min, and then with 1% BSA/PBS at room temperature for 25 min to minimize non-specific staining. The tissue sections were then pre-treated with streptavidin solution and biotin solution at room temperature for 30 min respectively to avoid high background signals due to the endogenous biotin or biotin-binding proteins in the tissues. The sections were incubated at 4°C overnight with 1:2000 dilution of guinea pig anti-VP1 anti-serum, followed by incubation of 30 min at room temperature with 1:2000 dilution of biotin-conjugated rabbit anti-guinea pig IgG, H & L chain (Abcam). Streptavidin/peroxidase complex reagent (Vector Laboratories) was then added and incubated at room temperature for 30 min. Sections were counterstained with hematoxylin. Cells infected or uninfected by rosavirus C RASM14A were included as positive and negative controls respectively in each staining. Cells were fixed in chilled acetone at -20°C for 10 min before incubation with antibodies for staining. Color development was performed using 3,3'-diaminobenzidine and images captured with Nikon 80*i* imaging system and Spot-advance computer software.

### Ethics statement

The collection of rodent samples was approved by the Committee on the Use of Live Animals in Teaching and Research (CULATR), The University of Hong Kong (HKU) (CULATR 2284–10) and the Department of Health, the Government of the HKSAR under the Animals (Control of Experiments) Ordinance, Chapter 340 (10-580/581/582 in DH/HA&P/8/2/3 Pt.23 & 13-34/35/36 in DH/HA&P/8/2/3 Pt.47). The production of antiserum against VP1 of rosavirus C in guinea pig experiment was conducted according to Canadian Council on Animal Care (CACC) guidelines (2002) with the protocol approved by CULATR, HKU (CULATR 2489–11) and the Department of Health, the Government of the HKSAR under the Animals (Control of Experiments) Ordinance, Chapter 340 (11-584/585 in DH/HA&P/8/2/3 Pt.31). The mice challenge experiment was approved by CULATR, HKU (CULATR 2545–11) and the Department of Health, the Government of the HKSAR under the Animals (Control of Experiments) Ordinance, Chapter 340 (11-845/846 in DH/HA&P/8/2/3 Pt.33). The mice study was carried out in strict compliance with animal welfare regulations. The mice were anesthetized by fetanyl/fluanisone/diazepam during the whole experiment. Standard guidelines prescribed in Pain and distress in laboratory rodents and lagomorphs, Laboratory Animals 28, 97–112 (1994) were strictly followed and the well-being of animals were monitored daily with a scoring sheet to ensure minimal pain and distress experienced by the mice.
